# The Challenges of Chinese University Leaders During the COVID-19 Pandemic Period: A Case Study Approach

**DOI:** 10.3389/fpsyg.2022.881969

**Published:** 2022-05-11

**Authors:** Xiudi Zhang, Xiaoming Tian

**Affiliations:** ^1^School of Education Science, Zhoukou Normal University, Zhoukou, China; ^2^School of International Studies, Shaanxi Normal University, Xi'an, China

**Keywords:** COVID-19, authoritarian leadership, challenges, uncertainty theory, case study

## Abstract

The outbreak of COVID-19 had a profound impact on the practice of university leadership in China. This study employs a case study as the research method, interviewing five Heads of the Departments from the Z University in China to examine the challenges to leadership in Chinese universities during the COVID-19 pandemic and explores effective countermeasures. Research findings reveal that the challenges they faced manifested in the government's closed management requirements and the students' demands for freedom of entry and exit, the dynamic and flexible disciplinary development and the rigid teaching evaluation, and big data-enabled governance and the habit of human experience-oriented management. In response to these challenges, this study proposes suggestions for the Z University leaders in the post-pandemic era: establishing rules and regulations with a relaxed degree, tolerating ambiguity in online teaching, improving the ability of intelligent technology, and taking opportunities to learn.

## Introduction

The outbreak of COVID-19 has had a profound impact on all sectors of society, out of which the education sector plays a special role and bears a great responsibility in fighting against the pandemic. In China, universities are often characterized by a high density of people. The majority of students' and teachers' daily routines are conducted in the form of collective activities (Yuan et al., 2018), which is very likely to cause cross-infection and transmission of COVID-19 and thus lead to social instability. Therefore, how to manage the universities has become a priority of local governments in pandemic management.

As a complex model of autonomous social organization, universities in China often experience a certain degree of uncertainty and ambiguity in both their processes and practices in terms of leadership (Liu, 2020). The sudden outbreak of COVID-19 has unavoidably exerted an influence on the leadership of Chinese universities by challenging their traditional way of governance and by slowing the momentum of the rapid development of higher education in China (Zhong and Nan, 2021). In the light of these conditions, this article aims to contribute to our understanding of Chinese university leadership in times of crisis, in particular during an ongoing pandemic, and highlights effective leadership approaches and implications for future practice.

Following the Introduction section, the current literature regarding crisis leadership and the capabilities required by leaders will be explored in the following section. The context for the study will be explained next, followed by the methodology. The findings are then presented according to the data and discussed with reference to previous research. Implications for leadership practice in universities in pandemic times are also suggested.

### Literature Review

The definition of a crisis as “an urgent situation that requires immediate and decisive action by an organization and, in particular, by the leaders of the organization” (Smith, 2012, p.58) fits with the situation facing Chinese university leaders when the universities were forced to close. In a crisis, leadership ability plays an important role. Smith (2012) highlight key leadership capabilities, such as strong interpersonal communication skills, the ability to synthesize information, the capacity to empathize and respect diverse perspectives, a capacity for optimism and flexibility, and the ability to capitalize on opportunities. Shingler-Nace (2020) identifies five elements of successful leadership during this crisis: staying calm, communication, collaboration, coordination, and providing support. DuBrin (2013) discusses leaders drawing on their emotional intelligence, including empathy and compassion, in times of crisis, and Kerrissey and Edmondson (2020) highlight the importance of leaders making themselves “available to feel what it is like to be in another's shoes” (p. 7). While the literature discussed so far all have some relevance for this study, this article elaborates on the leaders' abilities to capitalize on uncertainty for this study because of its specific relevance to the COVID-19 crisis in China.

Effective crisis leaders need to be comfortable with ambiguity and disorder. To some extent, the leaders need to adjust and improvise in acknowledging that mistakes will be made, and learning needs to be ongoing (Koehn, 2020). Koehn (2020) also highlights the COVID-19 crisis as “a powerful opportunity for organizations and teams of all kinds to better understand their weaknesses, what really engages and motivates their people, and their own reason for being” (p. 5). The importance of making rapid adjustments in direction or pivoting in response to COVID-19 and being prepared to learn has also been stressed (Johnson and Suskewicz, 2020). Earlier research also highlighted the importance of crisis leaders being adaptable and flexible as well as taking opportunities that crises create to make changes (Smith, 2012; DuBrin, 2013). This ability to capitalize on opportunities can involve learning both during and after crises (Deverell, 2011). While there is already considerable literature on leadership capabilities in crisis, there is a gap in the literature on principal leadership practices in a pandemic and in particular how leaders respond appropriately in the context of autonomous social organization.

### Uncertainty Theory

Uncertainty theory is developed and proposed to explain the behavior of uncertain phenomena, such as fuzziness and randomness (Liu, 2012). In organizations, there is even more reason to avoid uncertainties as they may endanger success (Kochenderfer, 2015). But at the same time, uncertainties are sought out as sources of innovation, and the ability to flexibly handle uncertainties becomes a competitive advantage (Kochenderfer, 2015). The importance of uncertainty for organizational functioning has long been recognized: “Uncertainty appears as the fundamental problem for complex organizations, and coping with uncertainty as to the essence of the administrative process” (Sun et al., 2019, p. 19). Uncertainty in governance structures is even more pronounced when there is an overlap in the powers of different organizational entities or when there are conflicts in the implementation of policies (Zhong and Yang, 2018). Take the principal responsibility system under the leadership of the party committee of a Chinese university as an example: although the party secretary and the president of the university seem to be a clear division of labor and cooperation, in the actual operation process, the boundaries between the two in terms of power, responsibility, and role are still, relatively, largely obscure.

Liu (2020) believes that the uncertainty of the university governance process is reflected in three areas: uncertainty in university decision-making, uncertainty in the execution of university decisions, and uncertainty of the effects of university decision-making implementation. Therefore, this article took the three areas of uncertainty as a theoretical observation tool and adopted the case study method to explore, analyze, and explain the dilemma and the path of resilient management in the process of the COVID-19 pandemic.

## Methodology

To gain more nuanced, inclusive, and detailed perspectives on the decision-making processes of leaders in Chinese universities during the COVID-19 pandemic, a qualitative case study design was employed for this study. The research method adopted *naturalistic* strategies that paralleled how the university leaders act in the course of daily management routines during COVID-19, typically interacting with informants in a natural and unobtrusive manner (Lincoln and Guba, 1985; Rallis and Rossman, 2012).

First, case studies are a common way to do qualitative inquiry. Abercrombie et al. (1984) argue that because a case study cannot provide reliable information about the broader class, it is only useful in the preliminary stages of an investigation since it provides a hypothesis that may be tested systematically with a larger number of cases (p. 34). However, it is misleading to see the case study as a pilot method to be used only in preparing the study's surveys, because as Flyvbjerg (2001) points out, this ignores the fact that the case study produces the type of context-dependent knowledge and the possibility of epistemic theoretical construction.

Second, case study research is not sampling research, that is, a process of selecting units from a population of interest (e.g., people, organizations). The first obligation for the researcher is to understand this one case whether we study it analytically or holistically, culturally, entirely, or by mixed methods—but we concentrate, at least for the time being, on the case (Stake, 2005). Therefore, this research has drawn attention to the question of what especially could be learned about the case of Chinese university leadership and uncertainty management during the pandemic period—an instance in time and space.

### The Study Context

The Z University is an ordinary undergraduate university in Henan Province in central China, located in a historical and cultural city. The school was founded in 1973 and upgraded to an undergraduate institution in 2002 with the approval of the Ministry of Education. There are 25,000 full-time students, 1,613 faculty members, including more than 440 professors and associate professors, more than 1,200 teachers with doctoral and master's degrees, more than 100 part-time master tutors, and more than 60 outstanding experts, teaching teachers, academic and technical leaders, and outstanding teachers in Henan Province.

In China, the university implements the principal responsibility system under the leadership of the party committee. The Z University has 20 faculties and 63 undergraduate majors, covering nine major disciplines, including literature, science, engineering, law, history, education, management, economics, and art. We chose Z University as the research site because it was rated as playing a leading role in the four universities of this city in pandemic control and prevention by the municipal government.

### Participant Selection

To recruit suitable research participants, we first visited Z University and identified the five departments that had played an essential role in pandemic prevention and control, including the President's Office, the Security Department, the Logistics Department, the Academic Affairs Office, and the Science and Technology Department. We then purposively invited the leader from each of the five departments who was in charge of pandemic control and management as the interviewees. In this way, a research participatory sample of five leader interviewees was selected and then individually interviewed face-to-face. All the interview data were digitally audio-recorded with the informed consent of the interviewee, and the duration of each interview ranged from 35 to 45 min.

All the interviews were semi-structured, starting with some pre-designed questions, such as “How have you led the department in managing the outbreak of COVID-19?,” “What was (were) the biggest challenge(s) to your leadership in the pandemic management?,” and “Has/have there been change(s) in your leadership since the outbreak of COVID-19?” While the participants verbally shared reflections on events or personal views, we also made additional, spontaneous follow-up inquiries to elicit a more detailed explanation of what and how the respondent had experienced in a bid to collect their stories, thoughts, and feelings in detail (Creswell, 2018). At the end of the interview, the president gave the author a collection of documents detailing the management of Z University during the pandemic period.

A question may arise as to whether a sample of a small number of participants would represent an adequate sampling size. This is a very common question in quantitative sociology. However, Small (2009) notes that saturation rather than representation is more important in qualitative research. Here, saturation is defined as data adequacy and means collecting data until no new information is obtained (Guest et al., 2006). An adequate sample size in qualitative research is one that permits—by virtue of not being too large—the deep, case-oriented analysis that is a hallmark of all qualitative inquiry (Sandelowski, 1995). The low level of variation we sought in the sample, and the focus on relatively high levels of homogeneity, means that it was a significant sample size—especially when additional institutional documents had been included for verification purposes.

### Analysis Process

#### Text Encoding

In this article, NVivo 12, a qualitative data analysis computer software package produced by QSR International, was used to discuss the problems in the post-pandemic era management of Z University. The use of this software helped the research team to find the required research resources in the empirical data and summarize them more objectively. The coding of the interview data by the research group mainly included the following six specific steps. First, the empirical interview data obtained were imported into the *NVivo 12* software. Second, all imported data were analyzed and encoded one-by-one, and the parts which were highly consistent with the research topic and clearly expressed were classified into different sub-nodes, and each node was named based on the research experience. Third, we temporarily put statements that did not match the research topic of this article or were answered ambiguously by the respondents into the free node. Fourth, after all the text encoding was completed, we used *NVivo 12* to query all the text content under the specific node and read the classification results. If necessary, the name of the node, the dependency relationship, and the logical schema had been corrected and deleted. Fifth, we focused on highly repetitive text content during the encoding process. The more frequently the text appeared, the more it reflected the universal significance of the respondent's psychological behavior. Sixth, we completed the coding after combing and induction.

#### Text Analysis

**Figure 1:**
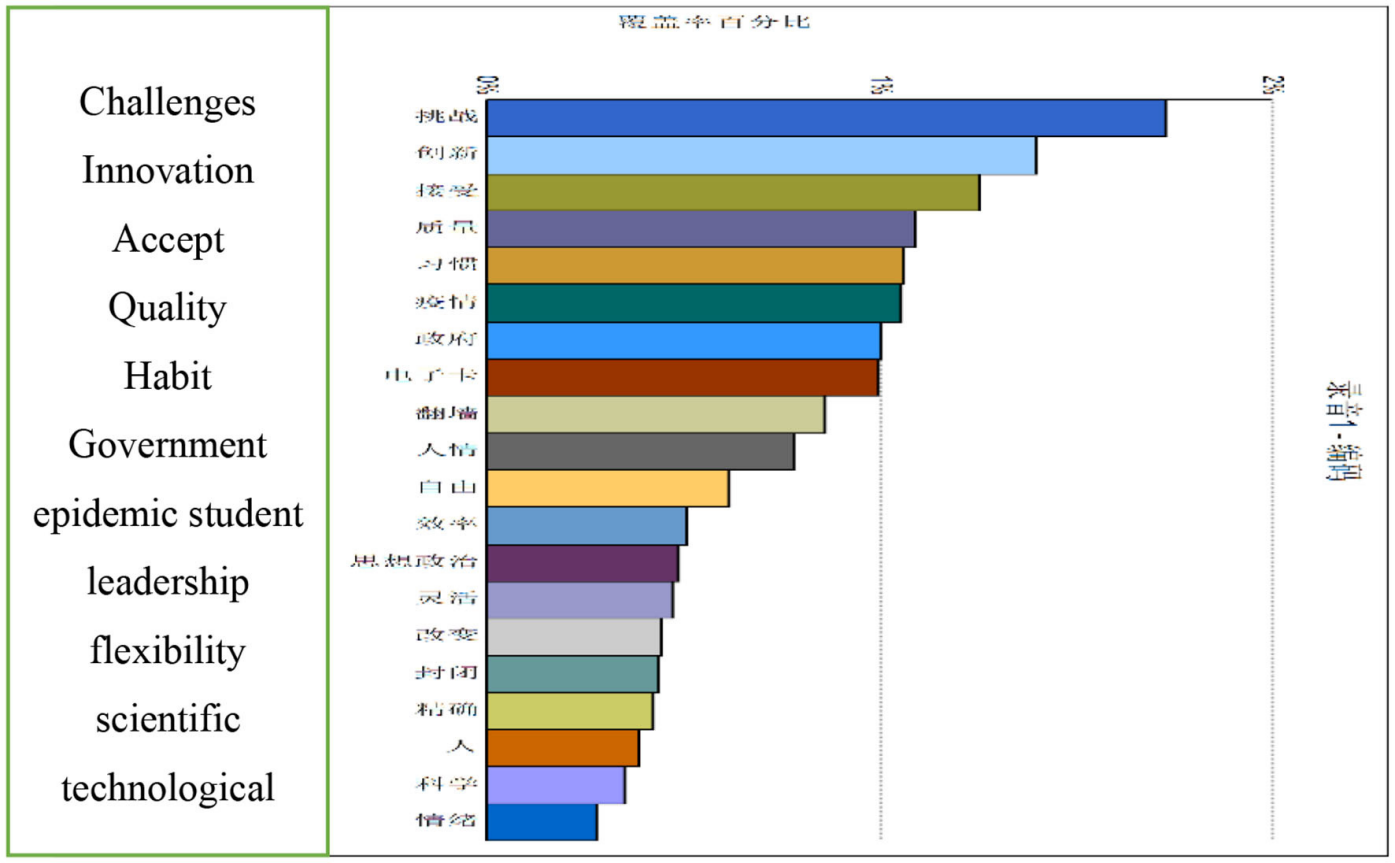
The percentage of the nodes.

The research group divided all coding variables into two categories: external factors and internal factors. These two classes were used as coding level one nodes, and the subsequent factors were level two. The secondary nodes of external factors included national, provincial government, and society, whereas the secondary nodes of internal factors included leadership, teachers, science and technology, and students. Through the scientific encoding of the text, 78 valid coding entries were finally obtained, from which the following 10 main nodes could be excavated: quality, government, pandemic, school, student, challenge, leadership, flexibility, scientific and technological information, and innovation. After the research group finally sorted the coding results, Figure 1 was obtained. From the data in Figure 1, it can be seen that in the process of pandemic management, the feedback from the five departments focused on the following aspects: the new management model, the requirements for innovation in the management functional departments, the acceptability of information technology empowerment management, the adaptability of online teaching quality monitoring and evaluation, and the data empowerment management compares to the habits of managers.

### The Legitimacy of the Study

The legitimacy of this study did not stem from the large amount of data collected, but from the sample topics obtained after scientific analysis of the experiences and ideas of a particular interviewee. This process of analysis was neither deductive nor inductive, but hypothetic (Creswell, 2018). Its purpose was to collect and analyze the narratives and views of the relevant pandemic departments of the Z University on pandemic management. This process of collecting and theorizing key data is known as a *systematic combination* (Fairbrother, 2013). Based on this, the cognitive patterns of these individuals rise to a common theme, and this purpose is to achieve the consistency of cognition in all aspects of the data collected (Stenhouse, 1977). Finally, all the topics were developed to illustrate management issues in the context of the pandemic at the Z University and to explore management strategies in the post-pandemic era.

## Findings

### Decision-Making in Campus Control

The social crisis caused by the coronavirus outbreak means that university administrators are facing rapid changes in scenarios, enormous psychological pressures, tough time constraints, and fragmented access to information. In this spatial-temporal context, if university administrators continue to use traditional management thinking to make decisions, it will lead to a rift between institutional supply and reality: a confusion in the normal management order of the school. Peter is the manager of the security department. He mentioned in an interview reviewing how to respond to an outbreak in a short period:

Before the outbreak of the pandemic, I was just transferred to this department. I did not feel any resistance to it. The crisis is new to everyone. I have to learn how to deal with it. I cannot get help from anyone as it is a new thing to everybody. They all “crossed the river by feeling the stones.” So, no matter what I do, it all makes sense.

The main job of Peter's department is to control campus access. The local government orders the local universities and colleges to close the campus when the cases of viruses increase. In China, the students live on campus. It means that nearly 25,000 students were locked down on campus during the pandemic period. This principle of campus access control helped to manage the flow of faculty members and students and to decrease the possibility of cross-infection with the virus.

The Z University enacted the principle of *the person who gives approvals is the person who takes charge of the campus access management*. That is, the leader of each department is responsible for controlling the physical flow of its faculty members and students and for reducing and minimizing any unnecessary arrivals or departures from the campus. However, it limited the freedom of students. According to the president of the Z University, Sam,

closed management is required from the government. However, there is no official interpretation available to the idea of “*closed*.” To reduce any possibilities of cross-infection of the virus, we adopted a strict campus control policy which has limited the freedom of students.

We furthered our inquiry to clarify the meaning of “*freedom*” mentioned by the leader and he replied to the question with great detail about the physical freedom of the students. Among the details that he offered, an example about beauty salons turned out to be very typical and apposite in explaining his views on students' freedom. According to Sam:

for university students, especially female students, they go outside the campus for hairdressing and nail salons before the university was closed. Now, the students were forbidden to leave campus during the pandemic. They cannot easily be satisfied with the only two salons on campus.

We inquired further about how the Z University dealt with this. Sam continued by saying:

According to students' psychology, first, we tried to find ways to clearly explain the reason to the students, so that the students can subjectively accept the state's control requirements, which is good for society, for everyone, and for individuals. The second is that we try to provide as many services as possible on campus, such as temporarily introducing more barbershops.

The interview data indicate that the university leader referred to the freedom of the students to their living needs. He comments that if students were locked down for a longer time, it would become a political issue.

Western countries may judge China to have no human rights if we closed the universities for a very long time. Western countries do not like wearing masks. Recently, in Xi'an city, students cried that “we want to go outside” after 11 pm in a university. Then someone posted it online so that the whole world knew that China restricts students' rights. It became a political issue. So, we would help students to realize that it is an ideological and political issue. The government and the university closed the university for your good, and for the good of society and this country. Second, we would satisfy their needs. Third, we would change our plan according to the situation, for example, we would let them go outside between 7 am to 8 pm when the cases reduce to zero in this city. (Sam)

Peter, the manager of the Security Department, responds with a similar view of students climbing over the wall:

Students' ideas are strange. When students were allowed to go outside the university, they would not go out to have a meal. However, the university now is closed. The students want to go out to have a meal. Some students have this mentality: in four years studying in university, it is a regrettable thing that none of them has yet climbed over the wall of the university to venture outside the campus (a way to show they are pursuing freedom). Even if they are caught by us, students still want to climb over the wall at least once.

In the interview, Lucy, the manager of the Logistics Department, could not understand why students wanted to climb over the wall. She further comments:

Teachers and students think, “there is no case of the virus in this city, why does the university not allow us to go out?” People on the outside also questioned, “why does the university not allow us to freely go to the university?” It is this emotion that spreads among each other. We have no way but to leave these problems unsolved.

### Execution of University Decisions of Online-Teaching

In pandemic prevention and control, the Z University adopted a dual plan model in teaching to ensure the effective operation of the university. The leaders required that online teaching should offer a replacement when classes could not be held normally in physical classrooms. In interviews, the manager of academics, Isaac, believed that the shift in teaching mode from offline to online has greatly improved the efficiency of the school.

By looking at the data that the system already has, we can see how many times each student submits the homework. Now the tasks assigned by the teacher can be reflected. On the online platform, we have the basic data of each student and lecturer's activity in the classroom. Of course, I can verify these basic data. I also know whether a teaching resource transmitted by the teacher is useful or useless for the course. Maybe the lecturer would upload some information just to improve his performance. I also can observe the lecturer's behavior. Therefore, to evaluate the course is very simple, we check the students' homework at the end of the semester.

The administrator believed that teaching evaluation becomes simpler, which underestimates the nature of complexity and changeability in academic evaluation. Later, according to the documents dealing with the prevention and control of Covid-19, this research group found that cameras were arranged in each classroom. It was convenient for the administrators of Z university to monitor a lecturer's offline teaching at any time.

### The Effects of University Decision-Making in Big Data-Assisted Management

The Z University required all staff and students to strictly abide by the *daily report* regulation. That is, all the in-service faculty members, including part-time ones, and the students were required to report their daily health data (whether they had a high fever, cough, fatigue, breathing difficulties, or other special symptoms, and their body temperature) to the university *via* a temporary data app developed by the university. Furthermore, the Z University arranged a special group of staff to collect data on vaccinations among the students and faculty members. The Z university aimed to get clearer vaccination information for pandemic control and to encourage those students and staff who have no contraindication to the vaccines to get vaccinated. However, it was very hard to popularize big data-assisted leadership before the outbreak of COVID-19, as expressed by the manager of the Science and Technology Department, Patrica:

It is our innovation to develop the app *Dingding* for pandemic control. The university leaders did ask us to invent the app at the beginning because it was too urgent for them to think of the idea, given that the outbreak of COVID-19 was very unexpected and sudden. What they asked us to do upon the outbreak was to do a brainstorm and think about what else we could do for pandemic control. Based on my very limited knowledge, the Z University was the first university in this region to apply big data-assisted apps on pandemic control.

On the positive side, the sudden outbreak of the novel coronavirus has driven the Z University's data-driven approach to COVID-19 governance.

Patrica: I think the big change is that there is an improvement in accepting the technology in management.

Question: Was it not accepted before the pandemic?

Patrica: It was not accepted.

Question: Are you sure?

Patrica: Yes, there was not much acceptance before. I thought it was due to inertia.

Question: What do you mean?

Patrica: Ideologically speaking, for example, if a department changed a younger leader, he may not have known that there was a better way to improve the efficiency of the management. If you suggested to him to use the data to collect the information, he would not be willing to listen and not be willing to learn and he would think you have changed his habits.

## Discussion

Several frameworks of crisis leadership were discussed in the earlier literature review. However, none seemed to adequately describe or explain the leadership approaches taken by the leaders in this study. In this section with reference to existing literature, three leadership practices, drawn from the three stages of decision-making presented in the findings, will be discussed. Implications for future crisis leadership practice will also be signaled. These practices have the potential to contribute to a new framework for Chinese university leadership in times of a pandemic and provide useful guidance for principals and other senior leaders in their responses.

### Establish Rules and Regulations With a Relaxed Degree

The manager's reference to “*crossing the river by feeling the stones*” and “*not being able to get help from others*” reflects the nature of crisis decision-making. Under enormous time and psychological pressure, managers' decisions often stem from intuition, preferences, and experiences (Liu, 2020). Peter's reaction to the crisis is to solve the pandemic problem by rules. Therefore, he set up the rules contained in the *Pandemic Prevention and Control Work Plan* (the 6^th^ edition). Reasonable rules and regulations contribute to the clarity and standardization of university management. University governance may seem chaotic, disorganized, and in a state of *anarchy*, but it is *organized* after all (Zhong and Yang, 2018). In the rules, students were required to stay at the university. However, the construction of the system must enable a leeway, and it is necessary to seek a combination of a rigid system and flexible management.

Students climbing over the wall occurred because of the lack of a flexible response to student freedom in the rigid closed management of the Z University. The strict closure of the school led to students' depression and inconvenience, resulting in the occurrence of this adverse event. Rigid management pursues a strict system and strengthens the constraints on teachers and students to meet the requirements of the government. However, if flexible management is integrated into it, it is more scientific and reasonable. Flexible management focuses on the humane care of teachers and students, giving emotional warmth and care, in order to improve the sense of belonging of teachers and students to the school, rather than ignoring the repeated appeals of students.

Considering the particularity of the pandemic, the change of management mode should be adjusted according to the development trend of the pandemic, that is, the adjustment from the *wartime state* of the pandemic to the *normalization* and back to the *wartime state* of the pandemic, and the change should be based on the repeated changes of the pandemic. This requires that the rules and regulations of the university be relaxed. Giesecke (1991) points out that in an organized anarchy institution, leaders sometimes need to break the rules and regulations to increase the flexibility and innovation of the institution. Therefore, university leaders need to have enough leadership to judge the situation, adapt to changes, and break the rules.

### Improve the Ability of Intelligent Technology

The internal organization of the university itself is huge and complex, coupled with the impact of the pandemic environment, which puts forward a more severe test for the governance ability of university leaders. In response to the call of the state to launch big-data-empowered pandemic management, the Z University is transitioning from the traditional management based on personal experience to the management model of big data empowerment, so university leaders should pay special attention to the training and cultivation of individual information literacy, and improve the individual's understanding and application ability of intelligent technology. In addition, previous research suggests establishing the awareness that data is the lifeline of decision-making in a situation of uncertainty and forming a research team of specialized institutions with data literacy to achieve common governance of education is vital (Lan, 2021). Whether constrained by the pandemic or other factors, university leaders should use their professional sensitivities to strengthen problem awareness, improve data literacy, and transform business problems into data problems to understand the complex issues in university governance in a more comprehensive, proactive, and systematic level.

### Tolerating Ambiguity in Online Teaching

Evaluation is an exploratory activity with typical uncertainty and complexity characteristics. The quality of teaching simply cannot be guaranteed exactly by the completeness of the instructional videos watched by the students, or by the frequency with which the teacher uploads the materials. Therefore, the production of knowledge requires universities to establish a relatively loose organizational structure, and the evaluation of courses should be dynamic and scientific. The lack of ideological awareness of teaching quality by the teaching administrators of Z University, the closeness of the teaching governance environment during the pandemic period, and the rigid teaching management at the subject level that does not conform to academic laws. Therefore, the Z university should consider preparation for future crises as well as taking opportunities to reflect on what could be changed and embracing different perspectives in a bid to maximize learning.

On the contrary, in the face of the high complexity and uncertainty of the external environment, tolerating ambiguity in university governance can bring to the university more flexibility, including more growth for the future development of new things. In this way, university decision-makers can have more autonomy and make better decisions. James (2006) points out that the university should be an emotional, relationship-focused organism, not a calculating, rational mechanical device. Therefore, the Z university should highlight the essential attributes of knowledge production, actively exert the autonomy of discipline construction, and mobilize the enthusiasm of teachers to participate in discipline governance.

### Limitation and Strength

There might be one perceived limitation that the argument of the article is generated from such a small sample size of a case study with only five research participants given that the case study merely portrays a single instance locked in time and circumstance of the Z University and is thus essentially conservative. As, Smith (2018) argues, generalizability is often identified as a limitation or weakness of qualitative research with smaller sample sizes. However, we argue that the small research sample might also be a research strength. A small qualitative sample enables us to research the pandemic governance practices of the five leaders from Z University in rich detail and to provide “a thick description or depth of understanding” (Palinkas, 2014, p. 851). This is also one of the distinct strengths of qualitative research—to gain in-depth insights and understanding of humankind, social materiality, and life phenomena.

## Conclusion

This small-scale study explored the perceptions of the Z University in the pandemic of COVID-19 on their leadership practices. This study contributes to our understanding of leadership in a pandemic by highlighting three areas of leadership practices that the president and others in leadership roles may wish to reflect on. These are preparing for crises by establishing rules and regulations with a relaxed degree, tolerating ambiguity in online teaching, improving the ability of intelligent technology, and taking opportunities to learn at all stages of the crisis. All these practices will assist in strengthening trusting relationships within the university, increasing their ability to recover. Many of these practices are also relevant to everyday leadership practice and it is to be hoped that, despite the challenges posed by the COVID-19 pandemic, opportunities will be taken to strengthen leadership practices.

## Data Availability Statement

The original contributions presented in the study are included in the article/supplementary material, further inquiries can be directed to the corresponding author.

## Author Contributions

XZ contributed to the conception, design of the study, organized the database, performed the statistical analysis, and wrote the first draft of the manuscript. XT validated the statistical analysis, wrote and revised sections of the manuscript, contributed to manuscript revision, read, and approved the submitted version. Both authors contributed to the article and approved the submitted version.

## Funding

This work was funded by the projects of New coronavirus and crisis management in colleges and universities of Henan Province (Item Number: 2021-ZZJH-509) and the the project of Introducing high-level talents in Zhoukou Normal University (Item Number: ZKNUC2019010).

## Conflict of Interest

The authors declare that the research was conducted in the absence of any commercial or financial relationships that could be construed as a potential conflict of interest.

## Publisher's Note

All claims expressed in this article are solely those of the authors and do not necessarily represent those of their affiliated organizations, or those of the publisher, the editors and the reviewers. Any product that may be evaluated in this article, or claim that may be made by its manufacturer, is not guaranteed or endorsed by the publisher.
